# Phase II study of TAS-106 in patients with platinum-failure recurrent or metastatic head and neck cancer and nasopharyngeal cancer

**DOI:** 10.1002/cam4.79

**Published:** 2013-04-18

**Authors:** Anne Tsao, Edwin Pun Hui, Rosalyn Juergens, Shanthi Marur, Tan Eng Huat, Goh Boon Cher, Ruey-Long Hong, Waun Ki Hong, Anthony Tak-Cheung Chan

**Affiliations:** 1 Department of Thoracic and Head and Neck Medical Oncology, University of Texas M.D. Anderson Cancer CenterHouston, Texas; 2 Department of Clinical Oncology, State Key Laboratory of Oncology in South China, Sir YK Pao Cancer Center, The Chinese University of Hong KongHong Kong; 3 Johns Hopkins University Sidney Kimmel Comprehensive Cancer CenterBaltimore, Maryland; 4 National Cancer Center SingaporeSingapore; 5 National University of Singapore HospitalSingapore; 6 National Taiwan University HospitalTaipei, Taiwan; 7 Division of Cancer Medicine, University of Texas M.D. Anderson Cancer CenterHouston, Texas

**Keywords:** Head and neck squamous cell carcinoma, nasopharyngeal cancer, TAS-106

## Abstract

TAS-106, a RNA polymerase inhibitor, was studied in solid tumors with potential clinical benefit and reasonable tolerability. We conducted a multicenter, international phase II trial of TAS-106 in salvage metastatic or recurrent head and neck squamous cell cancer (HNSCC) and nasopharyngeal cancer (NPC) patients. TAS-106 monotherapy was given at 6.5 mg/m^2^ over 24-h continuous infusion every 3 weeks. Translational studies for blood and tissue were included. Twenty-seven enrolled patients experienced the most common drug-related adverse events of neutropenia, fatigue, non-neutropenic fever, injection site reaction, and skin rash/dermatitis. The greater than or equal to grade 3 adverse events included neutropenia (14.8%), febrile neutropenia (7.4%), pneumonia (7.4%), and peripheral neuropathy (3.7%). The overall response rate was 0% in both subgroups; five HNSCC patients had stable disease (median duration 99 days) and four NPC patients had stable disease (median duration of 92.5 days). Median progression-free survival (PFS) for HNSCC patients was 52 days (95% CI 43.0–99.0 days) and 48 days (95% CI 41.0–83.0 days) for NPC. Median overall survival (OS) for HNSCC patients was 175 days (95% CI 92.0–234.0 days) and 280 days (95% CI 107.0–462.0 days) for NPC. The TAS-106 plasma levels were equivalent between Asian and Caucasian patients. There was no significant correlation of tumor UCK2 protein expression levels to TAS-106 efficacy. TAS-106 was reasonably tolerated in patients with platinum-failure HNSCC and NPC. The administration schedule of 24-h continuous infusion prevented neurologic toxicity, but had myelosuppression as its main toxicity. There was no anti-tumor efficacy seen with TAS-106 monotherapy. Future studies will focus on TAS-106 combinations and mechanisms of drug resistance.

## Introduction

In the United States, it is estimated that over 53,640 new cases of head and neck cancers will occur in 2013 with a corresponding 11,520 deaths [1]. The International Agency for Research on Cancer reported in a 2008 estimate that over ∼500,000 new cases of head and neck cancers and 120,000 new nasopharyngeal cases occurred worldwide [2]. In patients with metastatic advanced head and neck squamous cell cancers (HNSCC) and nasopharyngeal cancers (NPC), the treatment options are limited to systemic therapies. In the front-line metastatic setting, most HNSCC patients will receive platinum-5-fluorouracil (5-FU), platinum-taxanes, platinum-gemcitabine, or platinum-5-FU-cetuximab [3–6]. Metastatic nasopharyngeal patients are typically offered platinum-taxanes or platinum-5-FU [3, 7, 8]. Subsequent salvage therapies are limited and novel agents with new mechanisms of action are critically needed.

TAS-106 (Taiho Pharmaceutical Co., Ltd., Tokyo, Japan) (1-[3-C-ethyl-β-ribo-pentofuranosyl/cytosine, ECyd]) inhibits ribonucleic acid (RNA) synthesis by blocking RNA polymerases I, II, and III [9, 10]. TAS-106 has different properties from 5-FU as 5-FU incorporates into RNA and inhibits processing [10]. TAS-106 is processed after phosphorylation by uridine/cytidine kinase 2 (UCK2) into an active metabolite, ECyd-triphosphate (ECTP) [11, 12]. UCK2 is preferentially expressed in tumor cells compared with normal cells and higher activity of UCK2 has been correlated with TAS-106 sensitivity in tumor cell lines [12, 13]. The anti-tumor mechanism of action from preclinical models is believed to involve activation of RNase L and lead to rRNA fragmentation [14, 15]. Kazuno et al. have reported in a 10-panel human tumor cell lines that TAS-106 treatment decreased 45S rRNA precursor levels [10]. Additional preclinical studies have shown that TAS-106 has anti-tumor activity in various human cancer cells [9, 16], specifically HNSCC [17] and NPC [18].

Four early clinical trials have assessed the safety and tolerability of bolus and infusional schedules for TAS-106 [19, 20]. Hammond-Thelin et al. [20] reported the average terminal elimination half-life (*t*_1/2_) as 11.3 h and mean half-life between 8.2 and 13.8 h. A prior phase 1 study (9904) of TAS-106 monotherapy in solid tumor patients established the recommended dose of TAS-106 as 6.85 mg/m^2^ over 24 h as continuous infusion on day 1 of each 3-week cycle [17]. This 24-h continuous infusion schedule of administration was shown to have higher TAS-106 patient exposure compared with an intravenous push [20] or 4-h continuous infusion evaluated in other phase 1 studies [17]. In the TAS-106-9904 study, the dose-limiting toxicity (DLT) of 6.85 mg/m^2^ was greater than or equal to grade 3 neurological toxicity that lasted for 8 days [17]. At the higher dose of 8.56 mg/m^2^ the DLTs were grade 4 neutropenic fever and grade 4 neutropenia. The recommended phase II dose for TAS-106 was 6.5 mg/m^2^ by 24-h continuous intravenous infusion once every 3 weeks. The dosage was slightly reduced to 6.5 mg/m^2^ because 6.85 mg/m^2^ was the highest dose that was associated with a tolerable rate of DLTs in TAS-106-9904 study [17].

In the TAS-106-9903 trial, 6 of 18 patients had disease stabilization with a median duration of 103.7 days. At the suggested phase II dose, 2 of 8 patients had disease stabilization for a mean duration of 132 days. In the TAS-106-9904 trial, 14 of 28 evaluable patients had disease stabilization as a best response, but none of the patients on the study had a complete response (CR) or partial response (PR) [17]. This trial included four head and neck cancer patients and three of these patients had disease stabilization.

Based on these prior phase 1 studies and preclinical models, the phase II clinical trial in HNSCC and NPC patients was designed with the TAS-106 given at 6.5 mg/m^2^ per dose over 24-h continuous infusion. Translational correlates to assess pharmacokinetics, tumor tissue UCK levels, and whole blood RNase activity were included in the trial.

## Patients and Methods

This was an open label, multicenter, international phase II study to evaluate TAS-106 monotherapy in patients with recurrent or metastatic HNSCC or NPC. There were six sites that participated: two U.S.A., two Singapore, one Hong Kong, and one Taiwan.

### Patient eligibility

All patients had histologically confirmed HNSCC or NPC, recurrent locoregional and/or distant metastatic disease which were not suitable for local therapy, and had platinum-failure disease defined as (1) progression or recurrence during or within 12 months of receiving a platinum-based regimen as part of metastatic/recurrent disease or as primary curative intent treatment (concurrent platinum-based chemotherapy combined with radiotherapy as definitive therapy or postoperation adjuvant or neoadjuvant platinum-based chemotherapy). Patients were also required to have measurable disease by RECIST criteria, age ≥18 years at study entry, Eastern Cooperative Oncology Group (ECOG) performance status (PS) 0–2, hemoglobin >9.0 g/dL, platelet count ≥100,000/μL, absolute neutrophil count (ANC) ≥1500/μL, serum creatinine ≤1.5 mg/dL (133 μmol/L); if >1.5 mg/dL, a calculated creatinine clearance must be equal and more than 50 mL/min, total bilirubin ≤1.5 mg/dL (26 μmol/L), transaminases (aspartate aminotransferase [AST], alanine aminotransferase [ALT]) ≤2 times the upper limit of normal (ULN) (may be ≤5 times ULN if due to metastatic disease in the liver), and signed written informed consent per institutional and federal regulatory requirements. Patients were excluded for any radiographic or clinical evidence of brain metastases or leptomeningeal involvement, baseline peripheral neuropathy ≥grade 2, pregnancy or breast-feeding, prior radiation to over 30% of bone marrow (e.g., whole of pelvis or half of spine), or serious illness (congestive heart failure, uncontrolled angina pectoris, recent myocardial infarction, uncontrolled arrhythmias or hypertension, active infection, unstable diabetes mellitus, or psychiatric disorder that could interfere with consent or protocol compliance).

### Treatment plan

Each patient received TAS-106 at 6.5 mg/m^2^ intravenously over 24 h as a continuous infusion every 3 weeks. TAS-106 administration was continued until death, disease progression was documented, until an intolerable adverse event (AE) developed, withdrawal of consent, or until any other withdrawal criterion was met. Safety measurements included laboratory tests (hematology, chemistry, and urinalysis), vital signs, electrocardiograms, chest X-rays, physical examinations, and toxicity evaluations. Toxicities were evaluated at each course of therapy using the National Cancer Institute (NCI) Common Terminology for Adverse Events (CTCAE) version 3.0. Efficacy assessments occurred every two cycles or 6 weeks and included radiographic imaging with contrast-enhanced computed tomography (CT) scans of the head and neck, chest, and abdomen. Compliance was assessed by regular reviews of investigational product administration data including dosing recorded on case report forms, the investigational product accountability records and source documents reflecting patient's medical care.

Each TAS-106 4 mg vial was reconstituted with 4 mL of 0.9% sodium chloride for injection to produce a solution with a concentration equal to 1 mg TAS-106/mL. The reconstituted TAS-106 solution was further diluted with 0.9% sodium chloride for injection. The diluted TAS-106 solution (0.0036–0.244 mg/mL) was stable for 48 h at room temperature. TAS-106 was administered by 24-h intravenous infusion once every 21 days. Treatment with TAS-106 was repeated every 21 days (1 cycle). Administration of TAS-106 solution was completed within 48 h from the time of reconstitution.

Optional translational studies were included in this trial. This included pharmacokinetic (PK) assessments and whole blood collection for RNase level assessment from serially collected blood specimens during course 1. PK sampling for patients in Hong Kong, Taiwan, and Singapore was considered a mandatory procedure, but optional for patients in the United States of America.

### Methods and materials translational correlates

PK measurements obtained serial blood samples (4 mL/time point) during Cycle 1 at time points: 0 (predose), 2, and 4 h after starting infusion, 10 min before the planned end of infusion, 1, 4, 6, and 24 h after end of infusion. Whole blood samples (4 mL/time point) were drawn via venipuncture into a heparin-containing (green stopper) tube on Day 1 during Course 1. Whole plasma (approximately 2 mL) were transferred to a labeled cryotube and stored at −20°C for assay of TAS-106 until shipment to ADME/TOX Research Institute, SEKISUI MEDICAL Co., Ltd. (Ibaraki, Japan). Determination of TAS-106 concentrations in human plasma was performed as follows: human plasma (0.2 mL) was subjected to a solid phase extraction with ion-exchange mode, and the determination of TAS-106 was carried out by using a liquid chromatography/tandem mass spectrometry (LC-MS/MS). The lower limit of quantitation was 1 ng/mL of TAS-106 in plasma.

The tumor tissue studies focused on UCK2 messenger RNA (mRNA) and protein expression. The purpose of the gene expression investigation was to evaluate the relationship between expression of UCK2 in human tumor tissue and the efficacy of TAS-106. The mRNA expression levels were evaluated with quantitative real-time PCR and protein expression by immunohistochemistry. UCK2 phosphorylates the 3′-ethynyl nucleosides and enables the active metabolite to inhibit RNA polymerase [12, 21]. It is hypothesized that UCK2 cellular activity may be predictive for TAS-106 efficacy. In patients who volunteered for this optional analysis, fresh frozen tissue samples or formalin-fixed, paraffin-embedded (FFPE) tissue samples were obtained. For the tissue microdissection, six tissue slides were prepared from tissue blocks at a thickness of 5 microns each.

Total RNA was extracted using the Paradise® Plus Whole Transcript Reverse Transcription (WT-RT) Reagent System (MDS Analytical Technologies, Silicon Valley, CA). Total RNA quality and the molecular weight distribution of RNA were evaluated with Agilent 2100 Bioanalyzer (Santa Clara, CA) and quantity was determined by UV spectrophotometer (absorbance reading at 260 nm). RNA was diluted with Poly-i at a final concentration of 10 ng/μL (final volume 12 μL). RNA was converted to cDNA in duplicate reverse transcription (RT) reactions for each of the FFPE samples. cDNA for UCK2 and ACTB were amplified in a multiplex reaction for the purpose of increasing the quantity of the target cDNA prior to quantitative real-time PCR. Preamplification conditions consisted of 14 cycles using TaqMan® PreAmp Master Mix (2×), 180 nmol/L final concentration of primers for UCK2 and ACTB and 10 ng of cDNA for a final reaction volume of 50 μL. Relative levels of mRNA were analyzed using an Applied Biosystems (ABI, Foster City, CA) 7900 HTsequence detection system using TaqMan® chemistries. cDNA (1:20 dilution of preamplified template) 6.25 μL was amplified per reaction in triplicate wells for UCK2 and ACTB. UCK2 and ACTB primers and probes were provided by Taiho Pharmaceuticals Ltd. ABI master mix (2×) was added to each well for a final volume of 25 μL (primers final concentration 400 nmol/L, probe final concentration 100 nmol/L).

Standard immunohistochemical staining procedures for the protein analysis were applied and utilized the following antibodies: the primary antibody was anti-human UCK2 antibody (anti-hUCK2 rabbit IgG affinity purify No. 2, Taiho Pharmaceutical Co., Ltd., Tokyo, Japan). The secondary antibody was anti-rabbit peroxidase-conjugated IgG antibody (EnVision + System – HRP Labelled Polymer anti-Rabbit, Dako Japan Inc., Tokyo, Japan). The assessment for *H*-score was obtained as follows: *H*-score = Σ (staining intensity × % cells at each staining intensity).

### Statistical analysis

The primary objective was to evaluate progression-free survival (PFS) of TAS-106 in patients with recurrent or metastatic HNSCC or NPC. This study protocol predefined that clinically futile PFS median time was assumed to be lower than 3 months. According to the normal approximation formula of Lawless [5] to test the one arm survival time, the study would have 80–90% of statistical power to test the hypothesis that median PFS = 2 months versus the alternative that median PFS = 3 months >2 months with one-sided probability of 5%, when 37–52 evaluable patients have been accumulated with a 1-year recruitment and 1-year follow-up period, respectively. As there will be no interim assessment for PFS, no alpha spending was taken into consideration for the sample size calculation and the primary analysis will be performed at the 5% one-sided significance level. An interim efficacy analysis to evaluate the futility of TAS-106 for each subpopulation was conducted by the data monitoring committee (DMC). These analyses took place when approximately 13 patients, which correspond to a quarter of the total 52 patients, are accumulated in each subpopulation with an appropriate efficacy evaluation period (e.g., 3 months after the date of registration of the last patient), 50% of the planned study period has been completed (e.g., 3 months after the date of randomization of the last patient), and will estimate a conditional power for the final PFS analysis. In case the DMC does not conclude TAS-106 therapy as futile for both cancer types, an additional evaluable 13 patients with each cancer type will be accumulated that is a total of 52 evaluable patients. In the DMC concluded TAS-106 therapy as futile for both cancer types, further registration of patients with both cancer types will be terminated. In the advent where the DMC concluded TAS-106 therapy as futile for one of cancer type but not the other cancer type, further evaluable 26 patients only with latter cancer type will be accumulated that is a total of 39 patients with potentially effective cancer type will accumulated and evaluated by the end of this study. In the case where the DMC concluded TAS-106 therapy as futile for both of cancer types, further accumulation of patients will be terminated.

The PFS was calculated as days from the date of registration until the earliest date of documented disease progression, death, or censoring event. Secondary objectives included the overall response rate (ORR), best tumor response, overall survival (OS), safety, and tolerability. Clinical response was measured using the Response Evaluation Criteria in Solid Tumors (RECIST) guidelines every two cycles of therapy. Exploratory objectives were to investigate the relationship between selected biomarkers and efficacy and safety outcomes.

All data were processed and summarized using the Statistical Analysis System (SAS) (Version 9.1.3). Descriptive statistics and frequency counts were used to summarize efficacy and safety data. Median PFS was estimated using Kaplan–Meier method. Best tumor response was summarized by the categories of tumor response. Adverse events were coded and all AE summaries were based on the coded terms based on Medical Dictionary for Regulatory Activities (MedDRA) Version 14.0. AEs were summarized according to the numbers and percentages of patients reporting 1 or more occurrences during the study period. The summary statistics for the PK parameters were presented. Relationships of the pharmacodynamic (PD) variables to the PK assessment were examined.

The protocol, consent form, and any amendment were approved by the Institutional Review Board (IRB) which operates in compliance with the International Conference on Harmonization (ICH) guidance E6 (Good Clinical Practice [GCP]).

## Results

From 24 September 2008 until 26 October 2010, there were 27 patients enrolled and treated on the trial. In the NPC subgroup, 13 patients were enrolled and the preliminary data were analyzed to assess the safety and efficacy by the DMC members. With the result of the meeting, the enrollment of the NPC subgroup was terminated as of 10 June 2010 due to the lack of sufficient efficacy. For the HNSCC subgroup, 14 patients were enrolled and the trial was terminated as of 30 September 2010 due to lack of efficacy. Therefore, a total of 27 patients were enrolled and treated at six investigational sites; the U.S.A. (*n* = 12), Hong Kong (*n* = 9), Singapore (*n* = 5), and Taiwan (*n* = 1). The patient demographics are listed in Table 1. All NPC patients had undifferentiated histology and there were no World Health Organization (WHO) Type I NPC patients.

**Table 1:** Patient demographics

Characteristic	HNSCC, *N* (%)	NPC, *N* (%)	Total, *N* (%)
Number of patients enrolled	14	13	27
Median age (range)	56.5 (51–67)	53 (37–71)	57 (37–71)
Male gender	11 (78.6)	11 (84.6)	22 (81.5)
Ethnicity			
Caucasian	11 (78.6)	0	11 (40.7)
Asian	2 (14.3)	13 (100)	15 (55.6)
African heritage	1 (7.1)	0	1 (3.7)
ECOG PS			
PS 0	2 (14.3)	7 (53.8)	9 (33.3)
PS 1	10 (71.4)	6 (46.2)	16 (59.3)
PS 2	2 (14.3)	0	2 (7.4)
Staging			
Locally advanced unresectable	2 (14.3)	0	2 (7.4)
Distant metastatic	12 (85.7)	13 (100)	25 (92.6)
HNSCC primary site			
Oropharynx	7 (50)	N/A	N/A
Hypopharynx	1 (7.1)		
Oral cavity	3 (21.4)		
Larynx	3 (21.4)		
NPC WHO type			
Type I	N/A	0	N/A
Type II		1 (7.7)	
Type III		12 (92.3)	
Prior therapies^1^			
Chemo for metastatic disease	7 (50)	9 (69.2)	16 (59.3)
Adjuvant chemo	6 (42.8)	3 (23)	9 (33.3)
Neoadjuvant chemo	5 (35.7)	0 (0)	5 (18.5)
Geography participating centers			
U.S.A. (two centers)	12 (85.7)	0 (0)	12 (44.4)
Singapore (two centers)	0 (0)	5 (38.5)	5 (18.5)
Hong Kong	1 (7.1)	8 (61.5)	9 (33.3)
Taiwan	1 (7.1)	0 (0)	1 (3.7)

HNSCC, head and neck squamous cell carcinoma; NPC, nasopharyngeal carcinoma; ECOG, Eastern Cooperative Oncology Group; PS, performance status; N/A, not applicable.

1 Some patients had multiple lines of therapy.

### Compliance and toxicity

All the 27 patients received at least one dose of TAS-106 were included in the safety analysis. All the 27 patients received planned dose (6.5 mg/m^2^) of TAS-106 during course 1 and completed the same or reduced dosage in the following courses. Patients received a mean of 2.4 treatment courses and a median of two treatment courses (range 1–6). Doses were reduced following the courses. The percent of dose received divided by dose planned was higher than 95% between course 2 (22 patients) and course 4 (six patients). One patient completed a total of six courses. Three patients (two HNSCC and one NPC) withdrew consent during treatment after 101 days, 43 days, and 21 days on study.

Of the 27 patients evaluable for toxicity, all patients experienced at least one AE. The most common drug-related AEs (Table 2) were neutropenia (44%), fatigue (30%), non-neutropenic fever (22%), injection site reaction (26%), and skin rash/dermatitis (45%). Grade 3 and higher drug-related hematologic AEs included neutropenia (14.8%) and 2 (7.4%) febrile neutropenia. There were two cases of grade 4 febrile neutropenia (7.4%) and one case of grade 4 lymphopenia. The most common drug-related grade 3 or higher nonhematologic toxicities included pneumonia (7.4%), and peripheral neuropathy (3.7%).

**Table 2:** Adverse events by NCI-CTC version 3 grading scale

Adverse event	Grade 1, *N* (%)	Grade 2, *N* (%)	Grade 3, *N* (%)	Grade 4, *N* (%)	Grade 5, *N* (%)	Total AE, *N* (%)
Hematologic						
Anemia	0	3 (11.1)	0	1 (3.7)	0	4 (14.8)
Lymphopenia	0	0	0	1 (3.7)	0	1 (3.7)
Thrombocytopenia	0	1 (3.7)	0	0	0	1 (3.7)
Neutropenia	1 (3.7)	7 (25.9)	1 (3.7)	3 (11.1)	0	12 (44.4)
Febrile neutropenia	0	0	0	2 (7.4)	0	2 (7.4)
Nonhematologic						
Fatigue	5 (18.5)	3 (11.1)	0	0	0	8 (29.6)
Nausea	3 (11.1)	2 (7.4)	0	0	0	5 (18.5)
Vomiting	0	2 (7.4)	0	0	0	2 (7.4)
Diarrhea	3 (11.1)	0	0	0	0	3 (11.1)
Constipation	1 (3.7)	0	0	0	0	1 (3.7)
Anorexia	1 (3.7)	2 (7.4)	0	0	0	3 (11.1)
Abdominal pain	1 (3.7)	0	0	0	0	1 (3.7)
Ascites	0	0	0	0	0	0
Hyperbilirubinemia	0	0	0	0	0	0
Gastrointestinal perforation	0	0	0	0	0	0
Weight loss	0	0	0	0	0	0
Pneumonia	0	0	1 (3.7)	1 (3.7)	0	2 (7.4)
Non-neutropenic fever	5 (18.5)	1 (3.7)	0	0	0	6 (22.0)
Injection site reaction	3 (11.1)	4 (14.8)	0	0	0	7 (25.9)
Hemoptysis	0	1 (3.7)	0	0	0	1 (3.7)
Arterial thrombotic event	0	0	0	0	0	0
Venous thrombosis	0	0	0	0	0	0
Skin toxicity/rash	1 (3.7)	3 (11.1)	0	0	0	4 (14.8)
Dermatitis	8 (29.6)	0	0	0	0	8 (29.6)
Xerosis	2 (7.4)	1 (3.7)	0	0	0	3 (11.1)
Peripheral neuropathy	2 (7.4)	1 (3.7)	1 (3.7)	0	0	4 (14.8)
Other neurologic abnormality	3 (11.1)	0	0	0	0	3 (11.1)
Musculoskeletal pain or cramps	2 (7.4)	0	0	0	0	2 (7.4)

There were three patients who experienced grade 1–2 peripheral neuropathy, and one patient rated as having grade 3 toxicity. There were three patients with drug-related neurologic toxicity, primarily grade 1 dizziness. There was one incident of grade 3 somnolence that occurred on the study, but this was determined to be nondrug related as the patient had an aspiration pneumonia and respiratory insufficiency at the same time. No patients discontinued the study medication due to neurological toxicities.

There was only one (3.7%) patient who discontinued treatment due to possible drug toxicity. This patient received cycle 1 of TAS-106 and developed a non-neutropenic fever with pneumonia and significant decline in PS. The patient was removed from the trial due to the subsequent debility attributed to TAS-106. There were two reported grade 5 AEs which occurred in the same patient with a gastrointestinal perforation and concomitant aspiration pneumonia. This patient had received cycle 2 of TAS-106, and while the ANC was normal, she developed a small bowel perforation which led to sepsis with hypotension. On presentation, she was also found to have bilateral pneumonia and subsequently had respiratory failure which led to her death. The small bowel perforation and subsequent aspiration pneumonias were not considered to be drug-related events.

### Efficacy summary

Of the 27 patients enrolled on trial, one patient with HNSCC was not evaluable for response. The efficacy analysis was conducted separately for the HNSCC (*n* = 13) and NP (*n* = 13) subgroups. There was a 0% overall response rate in both subgroups; none of the patients had a CR or PR. Five (38.5%) patients with HNSCC had stable disease with a median duration of 99 days (range 42–168 days). Four (30.8%) patients with nasopharynx cancer had stable disease for a median duration of 92.5 days (range 85–100 days). For all patients with stable disease (*n* = 9), the median duration was 98 days (range 42–168 days). For the HNSCC group, the median duration on study was 43 days (range 26–168 days) and for the NPC group was 50 days (range 21–100 days).

**Figure 1:**
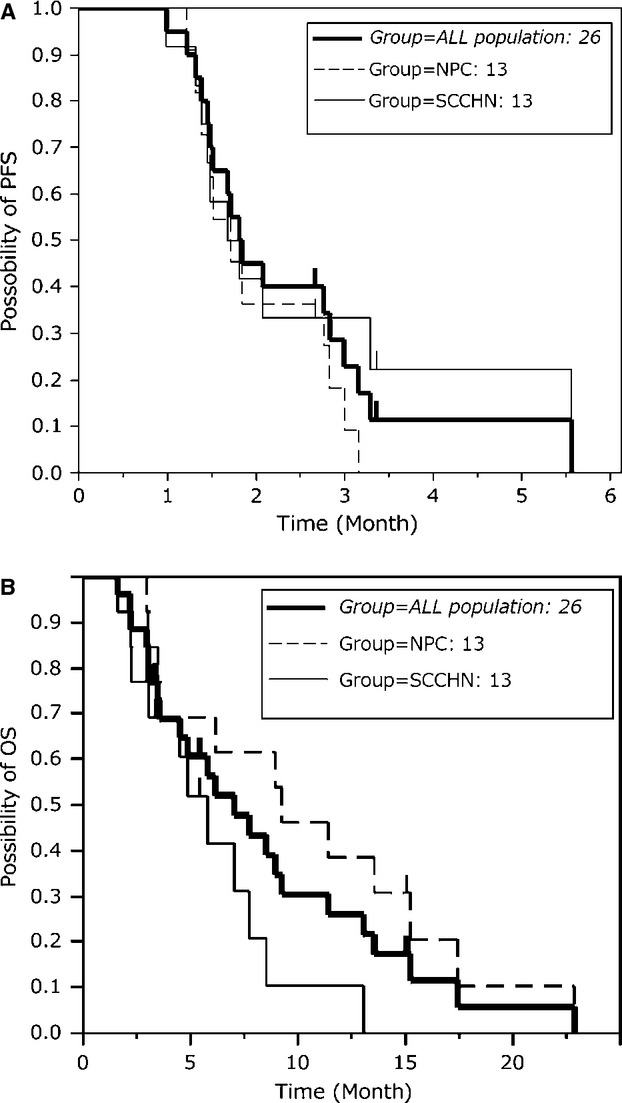
Survival outcomes for patients by (A) progression-free survival and (B) overall survival by tumor type.

For the primary endpoint (Fig. 1A), the median PFS for the HNSCC patients was 52 days (95% CI 43.0–99.0 days) and for the NP patients was 48 days (95% CI 41.0–83.0 days). For all the 26 patients analyzed, median PFS was 51 days (95% CI 43.0–83.0 days). The median OS for the HNSCC patients was 175 days (95% CI 92.0–234.0 days) and for the NP patients was 280 days (95% CI 107.0–462.0 days; see Fig. 1B).

### Exploratory studies

**Figure 2:**
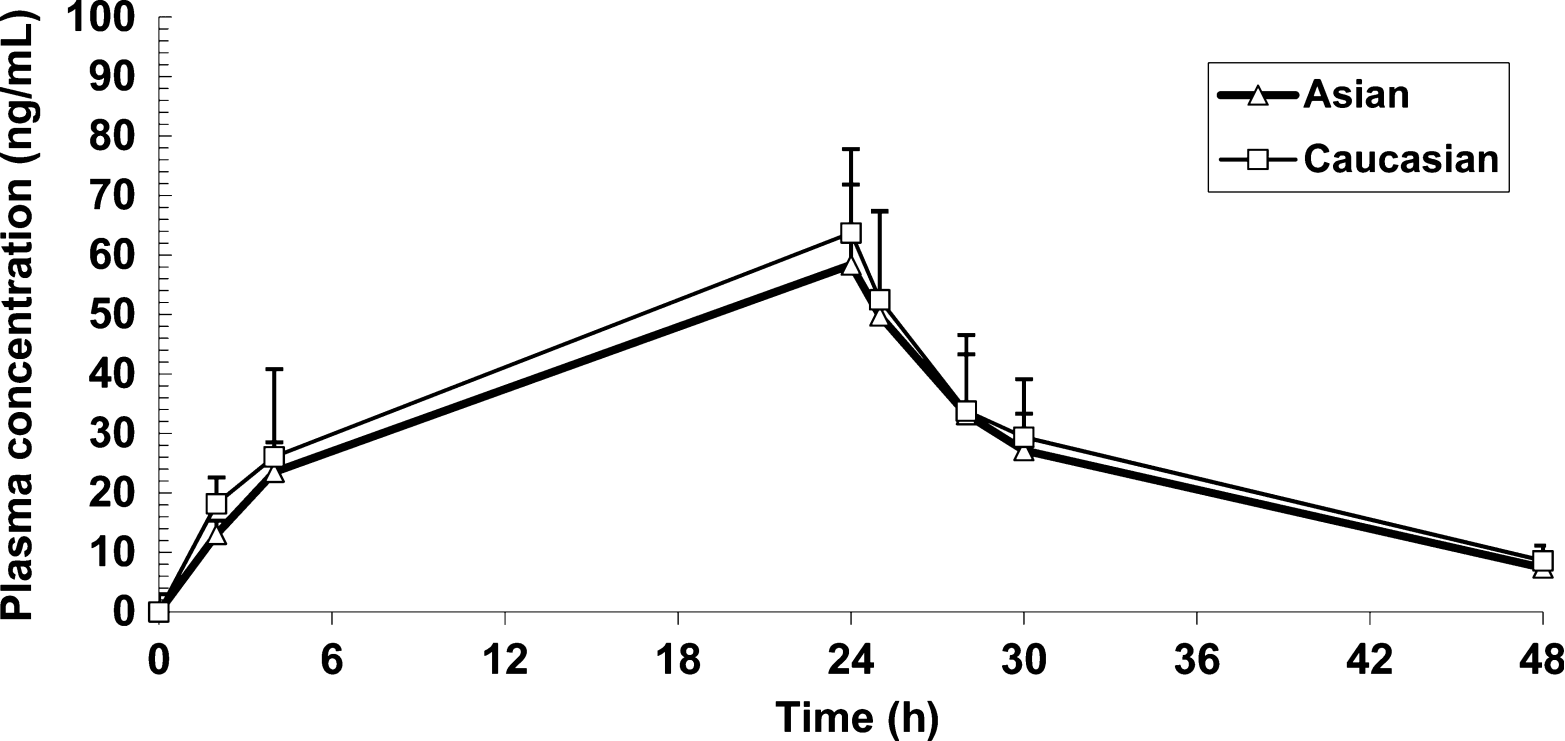
Plasma concentration plots of the first dose for five Asian patients and 11 Caucasian patients. Dose between 6.85 and 8.56 mg/m^2^ in TAS-106-9904 study was normalized to 6.5 mg/m^2^ to compare the ethnic differences.

From the PK assessments, TAS-106 concentrations in human plasma were assessed for the first dose of therapy in five Asian patients and 11 Caucasian patients (see Fig. 2). TAS-106 plasma levels were equivalent between Asian and Caucasian patients.

Additional translational studies were undertaken. To evaluate the mechanism of action, whole blood was assessed for ribosomal RNA (rRNA) levels after TAS-106 administration. In the majority of patients assessed (*n* = 24), there was no decrease in rRNA levels seen after administration of TAS-106.

The tissue translational studies were focused on determining the mRNA and protein levels of UCK2. A total of 12 patients consented to the optional procedures and their tissue samples were collected. One patient was not evaluable for protein expression level analysis; thus, the mRNA UCK2 expression levels for 12 patients and UCK2 immunohistochemical protein expression levels for 11 patients were measured. The H-scores for the 11 patients ranged between 0 and 170. There was no significant correlation of mRNA levels with tumor tissue protein UCK2 immunohistochemical levels. Of the 12 consenting patients, two patients (one HNSCC, one NPC) had high UCK2 protein expression levels with *H*-scores above 165. The NPC patient had SD as the best response for a duration of 87 days. The HNSCC patient with high UCK2 *H*-score had PD as the best response. There was no correlation between UCK2 protein expression levels in tumor tissue to response or duration of response to TAS-106.

## Discussion

In this international multicenter phase II study, TAS-106 was reasonably tolerated at a dose of 6.5 mg/m^2^ by 24-h continuous intravenous infusion once every 3 weeks in patients platinum-failure metastatic head and neck cancer or NPC. Unfortunately, there was no significant clinical activity seen by response rate and the primary objective of PFS was not met in either subgroup of patients. Although the original design was to enroll a larger number of patients, the interim efficacy analysis demonstrated a lack of sufficient clinical benefit and the study for both HNSCC and NPC patients was terminated after 27 patients were enrolled. Future development plans for this agent will consider combining TAS-106 with other therapeutic agents; as preclinical studies of TAS-106 and cisplatin have shown enhanced growth inhibition in tumor cell lines and murine xenograft models [22]. Preclinical models have also suggested that TAS-106 enhances the effects of radiation therapy, and may be a viable route of therapeutic development [23, 24].

In the toxicity analysis for TAS-106, the most common and problematic toxicities associated with the study medication were myelosuppression and infections. At the time the trial was designed, prophylactic growth factor support was not utilized on this trial as per standard of care. However, in total, there were eight patients that experienced grade 3 or higher infections during the trial and one patient was required to discontinue treatment. Although this group of patients had prior systemic therapy and often had comorbidities, future studies on this class of agent may need to consider the use of prophylactic growth factor support to limit myelosuppression to the patients.

One of the other safety concerns closely monitored on this trial was based on prior observations in the early phase I trials [19, 20] of significant peripheral neuropathy and neurologic AEs. In this phase II trial, there was only one patient who reported grade 3 peripheral neuropathy, which suggests that the 24-h continuous infusion schedule of administration was successful in limiting the amount of neurologic toxicity.

Unfortunately, due to lack of clinical activity on the 27 patients enrolled on this phase II trial, the translational correlates were very limited and this initial analysis failed to identify a predictive biomarker for TAS-106. There was no correlation of UCK protein tumor expression levels to clinical activity. Also, the whole blood RNase levels did not associate with any amount of disease stabilization. The potential mechanisms of resistance to TAS-106 require further elucidation. In vitro studies have suggested that diminished levels of intracellular ECTP, which is regulated by membrane transport receptors and UCK2 activity, are a mechanism of TAS-106 resistance [25]. Low UCK2 activity within tumor tissue would diminish or preclude metabolism of TAS-106 into its active form ECTP and decrease anti-tumor activity [11, 13].

This trial has several limitations, the first is the small sample sizes which occurred due to the interim analysis failing to demonstrate clinical activity with TAS-106 monotherapy. Secondly, the HPV-status on the HNSCC patients was not prospectively collected on this trial as the trial was designed and implemented prior to the common testing of HPV. Although it was not relevant in this negative study, it is nevertheless a limitation. Fianlly, as mentioned above the translational studies were not meaningful due to the lack of clinical activity of the monotherapy agent.

## Conclusion

TAS-106 was reasonably tolerated at a dose of 6.5 mg/m^2^ by 24-h continuous intravenous infusion once every 3 weeks in patients with platinum-refractory HNSCC and NPC. The administration schedule of 24-h continuous infusion therapy prevented neurologic toxicity, but had myelosuppression as its main toxicity. There was no anti-tumor efficacy seen with TAS-106 monotherapy. Future studies will focus on TAS-106 combinations and mechanisms of drug resistance.
